# MitoCarta2.0: an updated inventory of mammalian mitochondrial proteins

**DOI:** 10.1093/nar/gkv1003

**Published:** 2015-10-07

**Authors:** Sarah E. Calvo, Karl R. Clauser, Vamsi K. Mootha

**Affiliations:** 1 Howard Hughes Medical Institute and Department of Molecular Biology, Massachusetts General Hospital, Boston, MA 02114, USA; 2 Broad Institute, Cambridge, MA 02141, USA; 3 Department of Systems Biology, Harvard Medical School, Boston, MA 02115, USA

## Abstract

Mitochondria are complex organelles that house essential pathways involved in energy metabolism, ion homeostasis, signalling and apoptosis. To understand mitochondrial pathways in health and disease, it is crucial to have an accurate inventory of the organelle's protein components. In 2008, we made substantial progress toward this goal by performing in-depth mass spectrometry of mitochondria from 14 organs, epitope tagging/microscopy and Bayesian integration to assemble MitoCarta (www.broadinstitute.org/pubs/MitoCarta): an inventory of genes encoding mitochondrial-localized proteins and their expression across 14 mouse tissues. Using the same strategy we have now reconstructed this inventory separately for human and for mouse based on (i) improved gene transcript models, (ii) updated literature curation, including results from proteomic analyses of mitochondrial sub-compartments, (iii) improved homology mapping and (iv) updated versions of all seven original data sets. The updated human MitoCarta2.0 consists of 1158 human genes, including 918 genes in the original inventory as well as 240 additional genes. The updated mouse MitoCarta2.0 consists of 1158 genes, including 967 genes in the original inventory plus 191 additional genes. The improved MitoCarta 2.0 inventory provides a molecular framework for system-level analysis of mammalian mitochondria.

## INTRODUCTION

There is increasing appreciation for the essential roles that mitochondria play not only in oxidative phosphorylation and energy metabolism, but also in small molecule metabolism, ion homeostasis, immune signalling and cell death. Mitochondria originally descended from an endosymbiotic bacterium, predicted to resemble modern-day α-proteobacteria, early in eukaryotic evolution (1). Mammalian mitochondria contain their own genome (mtDNA), which encodes a total of 13 proteins that are all core components of oxidative phosphorylation. However, all of its remaining >1000 proteins (2) are nuclear encoded and imported into the organelle. Mutations in either the mtDNA or the nuclear genome underlie the largest collection of inborn errors of metabolism (3), and there is growing evidence that a gradual decline in mitochondrial activity is associated with aging and age-associated disorders.

**Figure 1.**
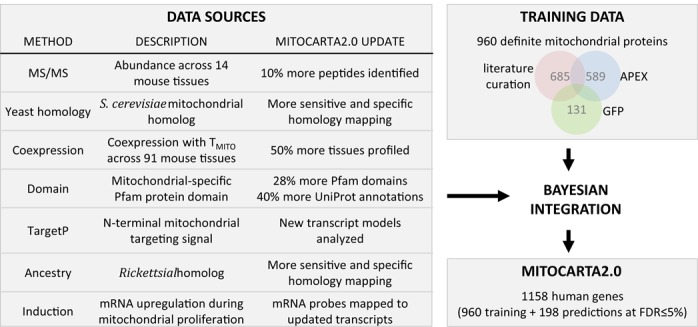
An updated inventory of mitochondrial proteins. MitoCarta2.0 is an inventory of 1158 human genes encoding proteins with strong support of mitochondrial localization. It is built by combining a large training set of 960 mitochondrial genes (based on literature curation, APEX-based mass spectrometry experiments and GFP-tagging/microscopy) with a Bayesian integration of seven genome-scale data sets, updated extensively from the previous MitoCarta1.0 inventory.

To fully understand the molecular basis of mitochondrial physiology and the organelle's role in disease, it is very useful to have a complete protein parts list for this organelle. In 2008, we constructed the MitoCarta1.0 inventory of mitochondrial proteins using multiple experimental and computational approaches (4). At that time, we purified mitochondria from 14 mouse tissues and performed in-depth tandem mass spectrometry (MS/MS) to identify mitochondrial proteins. We then compiled complementary clues of mitochondrial localization from homology to yeast and *Rickettsia prowazekii* proteins, presence of mitochondrial targeting signals and protein domains, and RNA coexpression across tissues and during mitochondrial biogenesis (Figure 1). Using a naïve Bayes integration (5), every mouse gene was assigned a combined score of mitochondrial localization from the seven data sources, each weighted by its accuracy based on large training sets of known mitochondrial and non-mitochondrial mouse genes. The resulting MitoCarta1.0 inventory of 1098 mouse genes contained 591 curated mitochondrial components used for training, 131 proteins validated using GFP/microscopy, and 376 proteins assigned to the organelle at a 10% false discovery rate (FDR). MitoCarta1.0 has been widely used to elucidate the function of uncharacterized genes and pathways (6–9), including reverse genetic screens (8,10) and forward genetic approaches to identify genes underlying rare mitochondrial disorders (11–14).

Here we present an updated MitoCarta2.0 inventory using the same overall strategy (Figure 1). The seven underlying data sources have been substantially updated using improved transcript models, MS/MS search algorithms, database versions and homology detection methods. Furthermore, the mitochondrial training set was increased by 60%. The MitoCarta 2.0 inventory consists of 1158 human genes and 1158 mouse genes encoding mitochondrial proteins. The MitoCarta2.0 website www.broadinstitute.org/pubs/MitoCarta freely provides the updated mitochondrial gene identifiers, evidence of mitochondrial localization, protein expression across 14 mouse tissues and protein sequences.

## INTEGRATION OF GENOME-SCALE DATA SETS

### Method overview

**Figure 2.**
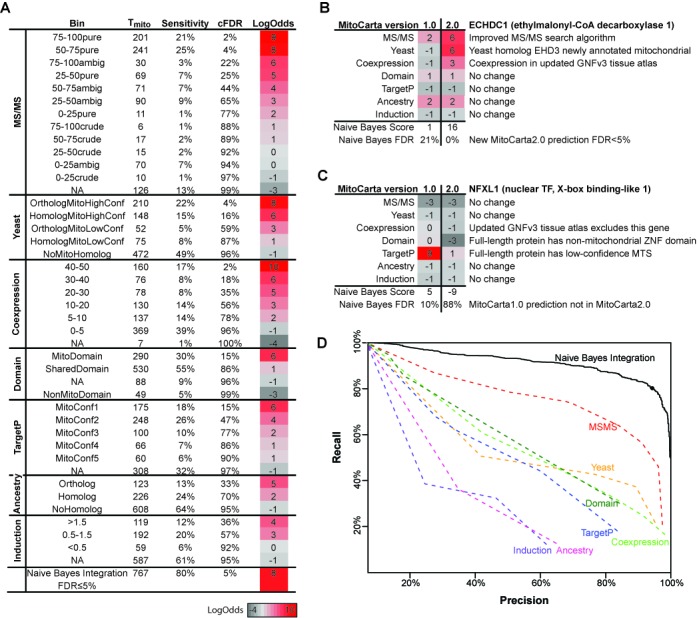
Naïve Bayes integration improves accuracy over individual data sources. **(A)** Accuracy of each of seven data sources and the Maestro naïve Bayes integration, calculated from the human *T_mito_* and *T_non_mito_* genes. **(B)** LogOdds scores for gene *ECHDC1*, in MitoCarta2.0 but not MitoCarta1.0, highlights improvements in three data sources **(C)** LogOdds scores for gene *NFXL1*, in MitoCarta1.0 but not MitoCarta2.0, highlights improvements to RefSeq gene models—as previous 58aa protein fragment XP_001052092 has been replaced with 918aa full-length protein NP_598682. **(D)** ROC curve shows accuracy of each data source individually as well as the combined naïve Bayes Integration. Black circle indicates 5% FDR threshold.

The MitoCarta2.0 inventory of mitochondrial proteins is constructed by first compiling the evidence of mitochondrial localization from seven complementary data sources (Figure 1). In parallel, we compiled large training data of known mitochondrial proteins (*T_mito_*) and non-mitochondrial proteins (*T_non_mito_*). These training data are used to assess the accuracy of each input data source by computing a likelihood score of mitochondrial localization at a range of input values (Figure 2). The seven individual likelihood scores are combined using a naïve Bayes methodology into an overall score for each gene (15). The resulting naïve Bayes score is far more accurate at scoring the known training data compared to each individual method (Figure 2). The final MitoCarta2.0 inventory is constructed by combining the *T_mito_* training data with all genes scoring below a 5% false discovery threshold (Figure 1).

### Gene models

The MitoCarta2.0 database is based on human and mouse RefSeq proteins (release 63) (16) that are mapped to NCBI Gene loci (ftp.ncbi.nih.gov/gene/DATA, 02/19/2014) (17).

### Training data

All human and mouse genes are partitioned into three sets: *T_mito_* (960 human, 961 mouse), *T_possible_mito_* (816 human, 750 mouse) or *T_non_mito_* (17468 human, 18918 mouse) as follows.

The *T_mito_* set of definite mitochondrial proteins is the union of (i) literature curation of proteins with strong experimental evidence of mitochondrial localization in mammals (see Supplementary Data), (ii) presence in the mitochondrial matrix proteome or intermembrane space (IMS) proteome in HEK 293T cells based on APEX-labeling (18,19) or (iii) confirmed mitochondrial localization by GFP-tagging and microscopy (4). 15 proteins in the previous *T_mito1.0_* were excluded based on updated literature curation (*Aadat, Armc4, Eln, Iqce, Mobp, Myl10, Nt5c3, Phyhipl, Pisd, Pla2g15, Pts, Tmem143, Tmem186, Tshz3, Txn1*). We include the APEX-based matrix and IMS proteomes in *T_mito_* given the extremely high specificity of APEX-labeling. Human and mouse *T_mito_* sets were created using human-mouse orthologs (best reciprocal BlastP hits, Expect < 1e-3) with the addition of species-specific genes with literature evidence.

Genes that did not meet our selective criteria for inclusion in *T_mito_* but which had some evidence of mitochondrial localization from the MitoP2 database (20) or the NCBI GO database (17,21) (downloaded 2/19/2014) were grouped into a *T_possible_mito_* gene set, not used for training.

We define the non-mitochondrial training set *T_non_mito_* as all genes not in *T_mito_* or *T_possible_mito_*. This differs from MitoCarta1.0, where *T_non_mito1.0_* contained 2519 genes whose proteins were reliably localized in non-mitochondrial compartments (e.g. ER, nucleus, lysosome, plasma membrane, vacuole). Thus*, T_non_mito2.0_* now contains thousands of cytoplasmic proteins that were previously underrepresented.

### Data integration

As in MitoCarta1.0, seven methods for determining mitochondrial localization were integrated using the Maestro naïve Bayes classifier whereby each method is weighted based on its accuracy (15). Training sets (*T_mito_* and *T_non_mito_*) were used to create a LogOdds score for each feature *F* at each predefined bin *b* (Figure 2A), defined as log_2_[*P*(*F_b_* | *T_mito_*) / *P*(*F_b_* | *T_non_mito_*)]. Assuming conditional independence between the data sets (Supplementary Figure S1), the individual LogOdds scores were summed to create a Maestro score for each gene (Figure 2B, C). For transcript or protein level scores, the gene inherited the highest score of any isoform. The scores for the seven genomic features were calculated at predefined ranges (Figure 2A) as follows (see Supplementary Data for details):

*MS/MS:* one of 12 categories corresponding to the percent of the protein [0–25%, 25–50%, 50–75%, 75–100%] detected by MS/MS peptides in mitochondria purified from mouse 14 tissues crossed with a subtractive proteomics enrichment score [crude-enriched, pure-enriched, ambiguous-enrichment], or NA if not detected (4). While the scoring method was identical to MitoCarta1.0, the original MS/MS spectra were searched against new RefSeq transcript models using updated SpectrumMill software that reversed faulty acquisition-time lock mass correction of MS1 scans and precursor masses, which resulted in 75% more spectra identified and 10% more unique peptides identified. The improvements are chiefly due to reversing the faulty lock mass calibration (see Supplementary Data).

*Yeast:* categorical score [OrthologMitoHighConf, OrthologMitoLowConf, HomologMitoHighConf, HomologMitoLowConf, NoMitoHomolog]. Homology was determined by BlastP (22) top hit (Expect < 1e-3) or jackHMMER (23) reciprocal hit (see Supplementary Data). Orthology was defined as a 1:1 homolog (i.e. the yeast gene had only one homolog in human/mouse). Genes with a yeast homolog/ortholog annotated as mitochondrial in SGD (Saccharomyces Genome Database, 03/06/14) (24) were scored as either MitoHighConf (SGD manual annotation, excluding dual localized proteins) or MitoLowConf (SGD dual localized proteins, or annotated mitochondrial based on high throughput data only). Genes that lacked a yeast homolog or where the yeast homolog was not annotated mitochondrial were categorized as NoMitoHomolog. Compared to MitoCarta1.0, this scoring method was more sensitive due to use of jackHMMER to identify distant homologs, and more specific due to the separate scoring of orthologs and homologs.

*Coexpression:* N50 score (number of *T_mito_* genes found within the gene's 50 nearest transcriptional co-expression neighbours, using Spearman correlation) within the GNF Mouse GeneAtlas V3 survey of gene expression across 91 mouse tissues (GSE10246) (25). In MitoCarta1.0, the GNFv1 atlas surveyed only 61 tissues (26).

*Protein domain:* categorical score [MitoDomain, NonMitoDomain, SharedDomain or NA] representing presence of a protein domain that is exclusively mitochondrial, exclusively non-mitochondrial, ambiguous or not present in any annotated eukaryotic protein (UniProt Knowledgebase Release 2014_06) (27,28). Protein domains were identified using HMMER (23) based on Pfam version 27 (29). This scoring was identical to MitoCarta1.0, with updated UniProt and Pfam databases.

*Targeting sequence:* confidence score of mitochondrial targeting signal from TargetP v1.1 (30), as in MitoCarta1.0.

*Endosymbiont ancestry:* categorical score [Ortholog, Homolog, NoHomolog], where homology was defined by BlastP (Expect<1e-3) or jackHMMER to *Rickettsia prowazekii* (see Supplementary Data), and orthology was defined as a 1:1 homolog (i.e. the *Rickettsia* gene has only one homolog in human/mouse). Compared to MitoCarta1.0, this scoring was more sensitive due to use of jackHMMER and more specific due to separate scoring of orthologs and homologs.

*Induction:* log2 fold-change of mRNA expression in cellular models of mitochondrial proliferation (overexpression of PGC-1α in mouse myotubes) compared to controls (15,31). Compared to MitoCarta1.0, probes in this data set were re-annotated using new transcript models (32), and data were normalized using gcRMA (33).

In contrast to MitoCarta1.0, these seven features were generated separately for all human genes and all mouse genes. Features that were mouse-specific (MS/MS, Coexpression, Induction) were mapped to human orthologs (BlastP best reciprocal hit, Expect<1e-3). The final MitoCarta2.0 lists for human and mouse were constructed as the union of all *T_mito_* genes and all Maestro predictions with FDR≤5%. As in MitoCarta1.0, FDR was defined as (1-SP)/(1-SP + SN x *O_prior_*) with specificity SP = TN / (TN + FP); sensitivity SN = TP / (TP + FN); *O_prior_* = 1500/21000; TP, true positives; TN, true negatives; FP, false positives; FN, false negatives.

### Accuracy of data sets and naïve Bayes integration

We assessed the accuracy of each data set and the combined Maestro naïve Bayes integration using recall and precision at predicting the training sets (Figure 2D). Recall is equivalent to sensitivity and precision is the percent of all predictions expected to be true (TP/(TP+FP) corrected for the size of the training data sets, equivalent to 1-FDR). As shown in Figure 2D, the Maestro naïve Bayes integration has substantially increased accuracy compared with the individual data sets. At the selected 5% FDR threshold on the human data set, the naïve Bayes method has 80% sensitivity and 99.6% specificity—far outstripping any single method. Using ten-fold cross-validation, the naïve Bayes integration showed a similar 79% sensitivity and 99.7% specificity at the same 5% FDR threshold.

## HUMAN AND MOUSE MITOCARTA2.0

**Figure 3.**
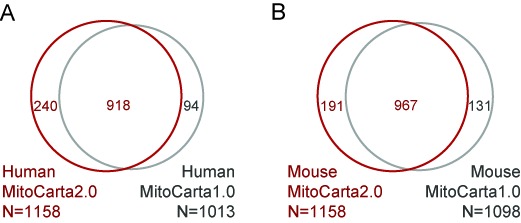
Overlap between MitoCarta versions

We separately performed a naïve Bayes integration to create a human-centric and mouse-centric MitoCarta2.0 inventory (Figure 3).

The human MitoCarta2.0 inventory contains 1158 genes, 79% of which overlap MitoCarta1.0 (Figure 3A). Of the 240 genes not in MitoCarta1.0, 100 were detected in the APEX-based matrix or IMS proteomes, 36 have other experimental literature evidence, and 104 achieve high probability of mitochondrial localization at the 5% FDR. For example, *ECHDC1* now has evidence of mitochondrial localization based on updated MS/MS, yeast homology and coexpression (Figure 2B). Of the 94 MitoCarta1.0 human genes that are now retired and not in MitoCarta2.0, 9 were pseudogenes no longer present in the latest RefSeq database and 85 score below our stringent 5% FDR. For example, *NFXL1* was in MitoCarta1.0 based solely on a high confidence TargetP prediction (Figure 2C), however the more recent RefSeq database replaces the previous protein fragment (XP_001052092) with a full-length protein (NP_598682) that has only a low-confidence TargetP prediction thus it is no longer predicted as resident in the mitochondrion.

The mouse MitoCarta2.0 inventory contains 1158 genes, 83% of which overlap MitoCarta1.0 (Figure 3B). 191 mouse genes were not in MitoCarta1.0, including 84 detected in the APEX-based matrix or IMS proteomes, 31 with other literature experimental evidence and 76 computational predictions (FDR≤5%). The previous version of RefSeq had a larger number of mouse pseudogenes. Of the 131 genes only in MitoCarta1.0, 42 were retired pseudogenes, 15 were *T_mito1.0_* genes no longer deemed to have strong evidence and rest were low-confidence computational predictions.

The vast majority of human and mouse MitoCarta2.0 genes are reciprocal top hits (96%). However, the separate inventories contain species-specific genes (e.g. human *ATAD3B*, mouse *Csl*) and predictions that had slightly different species-specific scores and thus exceeded the FDR threshold in only one of the two mammalian species (e.g. human *BOLA3, LDHB* and mouse *Ppm1m*).

## WEBSITE INTERFACE

MitoCarta2.0 inventory is available at www.broadinstitute.org/pubs/MitoCarta. The human and mouse mitochondrial inventories contain the Maestro naïve Bayes score and FDR, a summary of the evidence supporting mitochondrial localization and protein expression in 14 mouse tissues. Available for download are the naïve Bayes scores and mitochondrial evidence for all human and mouse proteins, BED files of gene coordinates, FASTA files of gene sequences and Excel files of the MS/MS peptides detected across 14 mouse tissues. Images supporting from previous GFP-tagging/microscopy experiments (4) are also available.

## COMPARISON TO OTHER MITOCHONDRIAL DATABASES

Multiple research groups have created inventories of mammalian mitochondrial proteins. To our knowledge, these include MitoCarta1.0 (4), MitoP2 (20,34–37), MitoProteome (38,39) and IMPI http://impi.mrc-mbu.cam.ac.uk/), however MitoP2 and MitoProteome are no longer available on the internet. Similar to MitoCarta, IMPI (Integrated Mitochondrial Protein Index) uses machine learning to predict mitochondrial localization in human, mouse, rat and cow based on experimental proteomics data in MitoMiner (40,41), antibody staining from the Human Protein Atlas (42) and mitochondrial targeting sequence prediction tools. The IMPI version Q2 2015 contains 1480 human Ensembl genes with substantial overlap with MitoCarta2.0 (980 in both, 500 IMPI-specific and 178 MitoCarta2.0-specific). Compared to MitoCarta's naïve Bayes methodology, IMPI's machine learning methods (support vector machines and random forests) have the advantage of allowing redundant data sets that are not conditionally independent, however the resulting scores are less readily interpretable and the techniques are more susceptible to overfitting of the training data. Additionally, IMPI does not provide the atlas of protein expression across tissues. Several other mitochondrial-focused web resources (43) aggregate useful mitochondrial data but do not include a reference set of mitochondrial proteins, e.g. MitoMap provides human polymorphism and mutation data (44,45), HMPDb (bioinfo.nist.gov/hmpd) aggregates data from nine knowledge bases and MitoMiner aggregates extensive proteomics data with MitoCarta1.0, UniProt and IMPI (40,41).

There are also many general databases of sub-cellular localization that provide breadth across many species and a hierarchy of subcellular locations. NCBI GO cellular compartments (21) annotates mitochondrial localization based on literature reports (including MitoCarta1.0). It currently includes over 1500 human genes linked to the mitochondrion (of which 1050 are in MitoCarta2.0), however it contains hundreds of genes with annotations electronically inferred from distant species or from single, controversial reports in the literature and furthermore there is no confidence score of mitochondrial localization. Similarly, UniProt (27,28) includes over 1094 human genes linked to mitochondria (of which 76% are in MitoCarta2.0) but lacks confidence scores of localization. COMPARTMENTS (46) lacks a downloadable list of mitochondrial genes, but for any query gene it provides a confidence score of localization to multiple cellular compartments (e.g. mitochondrion, nucleus, ER, cytoplasm) based on aggregating data from knowledge bases (e.g. UniProt), prediction algorithms (e.g. PSORT, yLoc) and text mining.

Overall, MitoCarta2.0 and IMPI provide the most specific inventories of mammalian mitochondrial components to the community, while broader databases such as NCBI GO and UniProt provide more breadth across species and cellular compartments.

## CONCLUSION

MitoCarta2.0 represents an easy-to-use inventory of mitochondrial proteins in mouse and human along with the evidence supporting mitochondrial localization for each protein—thereby providing a molecular framework for systematic studies of mitochondrial function and physiology. The MitoCarta database can be tuned to provide more or less stringent predictions of mitochondrial proteins by altering the FDR threshold. For example, when evaluating the results of a high-throughput screen of mitochondrial function, users may want to use a less stringent threshold such as 20% FDR. Similarly, when interpreting whole exome data from patients with mitochondrial disease, a 15% FDR might help interpret recessive mutations in genes with unknown function that may actually underlie mitochondrial dysfunction.

The current database has several important limitations. First, it is static and does not continually incorporate new literature evidence. Second, it is a mitochondrial-centric inventory that does not identify additional cellular localizations for proteins, or those that reside in the mitochondrion only under certain conditions. Third, it a gene-based inventory rather than an isoform-based inventory, because the underlying training data were available only for gene loci. Fourth, the training data were skewed toward proteins that reside within the double-membrane, thus it will be less accurate at predicting proteins of the outer mitochondrial membrane. Additional experimental data sets will be needed to interrogate the outer membrane and additional tissues and conditions not covered in MitoCarta2.0.

Despite these limitations, the updated MitoCarta2.0 mitochondrial inventory provides a valuable research tool to investigate mitochondrial pathways in health and disease. We expect that in the coming years this inventory will help elucidate the function of many specific pathways as well as to interpret many high-throughput data sets in molecular biology and human genetics.
